# Losartan improves alveolar bone dynamics in normotensive rats but not in hypertensive rats

**DOI:** 10.1590/1678-7757-2018-0574

**Published:** 2019-10-07

**Authors:** Gabriel Mulinari-Santos, Jaqueline Silva dos Santos, Letícia Pitol Palin, Ana Cláudia Ervolino da Silva, Cristina Antoniali, Leonardo Perez Faverani, Roberta Okamoto

**Affiliations:** 1 Universidade Estadual Paulista - UNESP, Departmento de Cirurgia e Clínica Integrada, Faculdade de Odontologia de Araçatuba, Araçatuba, São Paulo, Brasil.; 2 Universidade Estadual Paulista - UNESP, Departamento de Ciências Básicas, Faculdade de odontologia de Araçatuba, Araçatuba, São Paulo, Brasil.; 3 Brasil Affiliated with Research productivity scholarship (Process:306389/2017-7)

**Keywords:** Losartan, Spontaneously hypertensive rats, Bone, Hypertension

## Abstract

Hypertension is one of the main causes of premature death in the world; also, it is associated with several bone alterations. Preclinical studies have demonstrated delayed alveolar bone healing in hypertensive rats. However, losartan has been favorable for consolidation of bone grafts and reduction in active periodontitis. Therefore, losartan is suggested to be effective in bone formation stages, as well as in the synthesis of matrix proteins and mineralization.

**Objectives:**

To evaluate the alveolar bone dynamics in hypertensive rats treated with losartan by laser confocal microscopy and histological analysis.

**Methodology:**

Thirty-two rats, 16 spontaneously hypertensive rats (SHR) and 16 Wistar albinus rats, treated or not with losartan (30 mg/kg/day) were used. Calcein fluorochrome at 21 days and alizarin red fluorochrome at 49 days were injected in rats (both 20 mg/kg). The animals were submitted to euthanasia 67 days after treatment, and then the right maxilla was removed for laser confocal microscopy analysis and the left maxilla for histological analysis.

**Results:**

This study showed a greater calcium marking in normotensive animals treated with losartan in relation to the other groups. Laser confocal microscopy parameters showed higher values of bone volume formed, mineralized surface, active surface of mineralization and bone formation rate in normotensive animals treated with losartan. However, a smaller mineralized surface was observed in all hypertensive animals.

**Conclusion:**

Losartan can improve bone mineralization parameters under normal physiological conditions, but the same anabolic effect does not occur under hypertension.

## Introduction

Hypertension is a chronic disease with the highest number of premature deaths and care assistance in the world.^1^,^2^ Its high prevalence reaches about one in three young adults in the world, and this number is estimated to increase.^3^ In addition to cardiovascular diseases, hypertension is associated with chronic renal failure, stroke, and bone abnormalities, such as: abnormal calcium metabolism,^4^ altered alveolar bone quality,^5^ delayed alveolar bone healing,^6^ bone density loss,^7^ increased risk of fractures^8^ and, consequently, osteoporosis.^9^ The question arises whether bone changes may be associated with the vasoconstriction present in hypertension.^10^ Thus, plasma calcium supply would be reduced, impairing alveolar bone mineralization. Finally, alveolar bone dynamics can interfere direct in the success of clinical procedures, such as: tooth extraction, consolidation of bone grafts and implant osseointegration.

Renin-angiotensin system inhibition is a target for hypertension control.^11^ Then, angiotensin II receptor blockers can prevent vasoconstriction and high blood pressure.^12^ In addition, angiotensin II enhances bone resorption by increasing RANKL and osteoclastic activation.^6^,^13^,^14^ Also, it has effects of osteoblastic lineage changing,^15^,^16^ by decreasing the expression of osteogenesis-related transcription factors via the AT1 receptor, such as Runx2, Msx2, and osteocalcin.^15^ Losartan, as an angiotensin II receptor blocker,^17^ has attracted interest not only for being a vasodilator, but also for its positive effects in bone metabolism.^18^,^19^ Preclinical studies have reported benefits of losartan on bone fracture healing and reduction in bone fracture risk.^20^ They also reported improved bone graft healing.^10^ In *in vitro*^18^ and *in vivo* studies,^21^ losartan significantly enhanced bone density by decreasing osteoclastogenesis and increasing osteoblastic activity. Therefore, a topic to be evaluated referred to how antihypertensive drugs interfere in alveolar bone dynamics.

The action of losartan in the renin-angiotensin system, and its consequent influence on bone metabolism, is widely studied.^19^,^22^,^23^ Animals presented decrease in periodontal bone loss^22^,^23^ and orthodontic movement^19^ by effect of losartan in OPG/RANKL system. Other studies indicate delay in alveolar bone healing^6^ and lower alveolar bone density in spontaneously hypertensive rats (SHR).^24^ Vasoconstriction, with a consequential reduction in plasma calcium supply, can be the reason of delayed and decreased bone mineralization in hypertension. In this aspect, the vasodilatory effect of losartan can support alveolar bone mineralization.^10^ This hypothesis was tested in a study evaluating alveolar bone dynamics in hypertension rats treated with losartan. Despite the high prevalence of hypertensive patients, alveolar bone dynamics in hypertension remains uncertain.

Therefore, this study aimed to evaluate the areas of calcium marked by fluorochromes and alveolar bone dynamics parameters in hypertensive rats treated with losartan, such as bone volume formed, mineralized surface, active surface of mineralization, calcein, bone formation rate, and mineral apposition rate.

## Methodology

### Study design and ethics

This study received a favorable opinion from the Animal Research Ethics Committee under number 00404-2016. All work has been developed according to the ARRIVE guidelines. A total of 32 adult (16-week) male rats, whose body weight ranged from 250 to 300 grams, were used; being 16 Wistar (Rattus novergicus albinus Wistar) and 16 SHRs. The animals were kept in cages in a stable temperature environment (22°C ± 2°C, light control cycle 12 light hours, 12 hours dark), balanced diet (Ração Mogiana Alimentos SA, Campinas, Brazil). The animals were divided into four groups according to the drug treatment: Wistar, Wistar losartan, SHR, and SHR losartan. Randomization sequence of groups was performed using a computer-generated list of Stata 9.0 (StataCorp, College Station, TX, USA).

### Losartan treatment and systolic blood pressure

Losartan (Biosintetica, São Paulo, Brazil) was orally administered at a dose of 30 mg/kg daily until euthanasia.^10^ Animals in the untreated groups received only water in this period. The systolic blood pressure was measured every 7 days by tail-cuff indirect plethysmography using the Physiograph®, MK-III-S plethysmograph (Narco Bio-systems, Houston, Texas), performed according to previous studies.^6^,^10^ Losartan controlled all hypertensive animals' blood pressure, without changes in the Wistar group treated.

### Fluorochrome application

21 days after beginning drug treatment, the fluorochrome calcein was administered intramuscularly (20 mg/kg). Alizarin red fluorochrome was also used intramuscularly at a dose of 20 mg/kg for each animal after more 28 days, according to previous studies.^25^–^27^ The first injected calcein fluorochrome indicates calcium deposition in old bone and the subsequent alizarin fluorochrome in the newly-formed bone.

### Euthanasia

All animals were submitted to euthanasia 67 days after drug treatment with a intravenously lethal excessive anesthetic dose (60 mg/kg, Tiopental Cristália Ltda; Itapira, SP, Brazil). Therefore, this experimental period is adequate to characterize the modeling or remodeling process of old bone into new bone indicated by fluorochormes and histology.

### Laboratory processing

The right side of the maxilla was removed and fixed in 10% formaldehyde. After 48 hours, they were washed 24 hours in running water and dehydrated in an increasing alcohol sequence. The samples were soaked and infiltrated in a solution of acetone and methylmethacrylate MMAL (Classical, Dental Articles Classical, São Paulo, SP, Brazil) in ratio of 1:1, followed by methylmetracrylate baths. Benzoyl peroxide (1%, Riedel-de Haen AG, Seelze-Hannover, Germany) was added to the latter bath. Samples were placed in glass tubes and maintained at 37°C for 5 days. Resin blocks containing the samples were removed from the glass tubes after polymerization. The blocks were reduced to the maxilla sagittal plane. The reduction occurred with “Maxcut” drill mounted on an electric motor (Strong 210, São Paulo, SP, Brazil). Afterwards, the samples were submitted to a progressive wear on politriz (Ecomet 250 pro/automet 250, Buchler, Lake Bluff, Illinois) with sandpaper (120, 300, 400, 600, 800, and 1200 granules, Carbimet, Buchler, Lake Blunff, Illinois) under fluorescent light up to the thickness of 80 μm, conferred with digital caliper (Mitutoyo, Pompeia, São Paulo, Brazil). The sections obtained were mounted on slides immersed in mineral oil (Petrolato liquid, Maantecor, Taquara, Rio de Janeiro, Brazil) and fixed with enamel to prevent the oil from being trapped.

### Laser Confocal Microscopy

**Figure 1:**
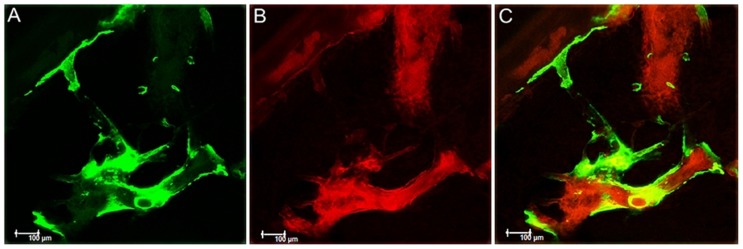
Alveolar bone dynamics by confocal laser microscopy. Representative image of the biomarking of the fluorochromes in the alveolar bone of the maxilla estimated by “Color Selection” tool in the Image J program (Processing Software and Image Analysis, Ontario, Canada). A: Green biomarking of calcein fluorochrome. B: Red biomarking of the alizarin fluorochrome. C: Overlapping of both fluorochromes characterizing the alveolar bone dynamics

The longitudinal scan of each maxilla was performed using Leica CTR 4000 CS SPE microscope (Leica Microsystems, Heidelberg, Germany), using a 10x objective (Original magnification of 100x). The area evaluated was the bone adjacent to the apical third of the right upper incisor. After selecting the thickness of the slices, images were reconstructed in microscope program (Leica CTR 4000 CS SPE, Leica Microsystems, Heidelberg, Germany). The blue filter was used to visualize the calcein fluorochrome, shown in green color (Figure 1A). The alizarin fluorochrome was revealed in red by green filter (Figure 1B).

Overlapping of the two layers of fluorochromes was performed, representing calcium deposition in both periods (Figure 1C). Therefore, this overlap shows the conversion of old bone into new bone. Images were saved in TIFF format and moved to the Image J program (Processing Software and Image Analysis, Ontario, Canada). Using the “color selection” tool, each image was standardized according to hue, saturation, and brightness. Both bone types were observed in the same setting on a single slide for measure the parameters: Bone volume formed, mineralized surface, active surface of mineralization (alizarin fluorochrome), calcein, bone formation rate, and mineral apposition rate (MAR).

All parameters were estimated using the Image J program (Processing Software and Image Analysis, Ontario, Canada), using calcein fluorochrome initial values and the active mineralization surface, according to previous study.^28^ Mineral apposition rate was the daily value of mineralization, estimated by the distance between calcein and alizarina, divided by the 28 days among their administrations. The mineralized surface was estimated by dividing calcein and alizarin percentage by the total bone surface area. Bone volume formed was estimated by the multiplication of MAR by the mineralized surface and the total bone surface area.

### Histological analysis

Later, the left hemi-maxilla was washed 24 hours in running water and decalcified in 10% EDTA for 6 weeks. They were washed for 24 h, dehydrated through an alcohol sequence, cleared in xylene and embedded in paraffin (Merck, Kenilworth, NJ, USA). 5-µm thick sections were cut with a microtome and mounted on glass slides. Alizarin red and Stevenel's blue-stained slides were captured using a Nikon microscope (Eclipse 80i, Shinagawa, Tokyo, Japan). Bone tissue morphology was qualitatively evaluated, establishing comparison between the groups. The slides were photomicrographs magnified from the originals by 10x.

### Statistical analysis

The GraphPad Prism 7.0 profiler (GraphPad Software, La Jolla, USA) was used for statistical test. Shapiro-Wilk test (p<0.05) was performed to verify homoscedasticity and whether the results were parametric. After confirming the normal distribution, Anova test was performed followed by Tukey post-test for multiple comparisons when necessary. For all tests, the p-value considered as significant was p<0.05.

## Results

### Systolic blood pressure and body weight

**Figure 2:**
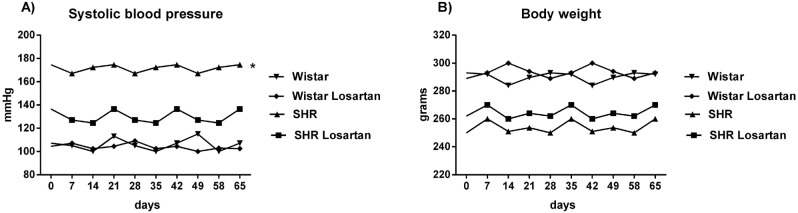
Systolic blood pressure and body weight. Graph with the systolic blood pressure measured by tail-cuff indirect plethysmography using the Physiograph®, MK-III-S plethysmograph (Narco Bio-systems, Houston, Texas). The * indicates statistical difference of the SHR blood pressure group compared with the others (p<0.05). Additionally, the body weights were checked during the experiment and were similar between the groups treated with balanced diet (Ração Mogiana Alimentos SA, Campinas, Brazil)

Figure 2A shows the systolic blood pressure along the experiment. Losartan controlled all hypertensive animals' blood pressure. SHR obtained the highest systolic blood pressure mean values with statistical difference compared with SHR Losartan (171.4±0.6 versus 129.3±0.9 mmHg, p=0.04). There was no variation between Wistar and the Wistar group treated (105.1±1.1 versus 104.2±1.0 mmHg, p=0.14). Body weights mean in Figure 2B also showed no statistical difference (p>0.05), Wistar=294.1±1.9 grams, Wistar Losartan=289.4±1.5 grams, SHR=253.6±1.1 grams, and SHR Losartan=264.2±1.3 grams.

### Qualitative analysis

**Figure 3:**
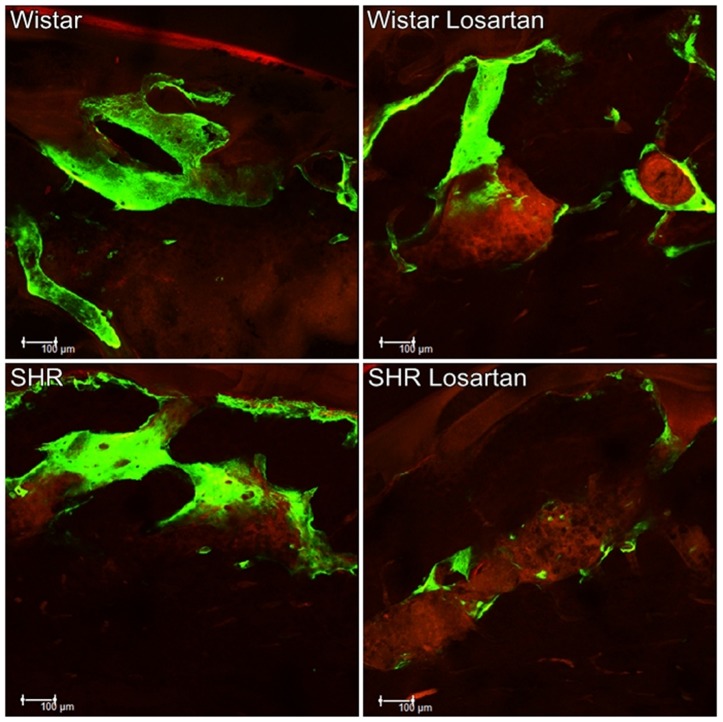
Alveolar bone dynamics. Representative image of bone dynamics by overlapping the fluorochromes of calcein and alizarin in the alveolar bone of the maxilla in each group for Wistar, Wistar Losartan, SHR, and SHR Losartan using the Image J (Processing Software and Image Analysis, Ontario, Canada)

Figure 3 shows the alveolar bone dynamics through the precipitation of fluorochromes in each group. The fluorochromes that bound to calcium at the time of their precipitation in organic bone matrix enabled the overlap between old (green) bone and new (red) bone. The first fluorochrome injected was calcein; therefore, it was the green biomarking of the old bone. Calcein biomarkers were present in all groups, with higher intensity in both Wistar groups and lowest in the SHR Losartan. The last injected fluorochrome was Alizarin, so the biomarking in red represented the newly-formed bone, which we call the active surface of mineralization. It was also present in all groups, being more intensive in the Losartan Wistar group and less evident in the SHR group.

### Quantitative analysis

#### Bone volume formed

**Figure 4:**
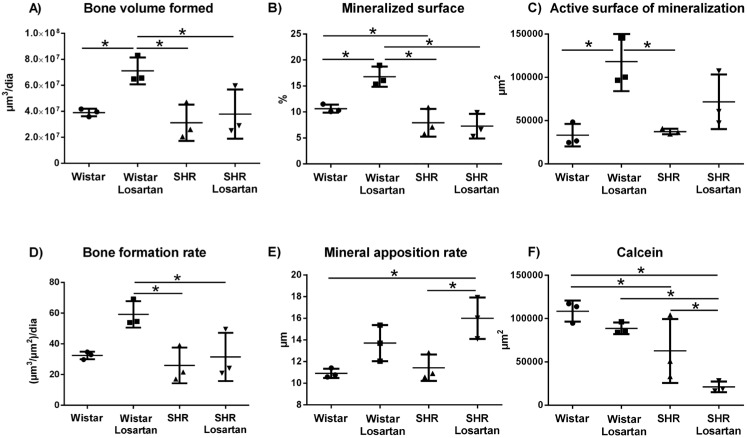
Parameters of laser confocal microscopy. Scatter-plots graphs with the confocal microscopy parameters evaluated in the alveolar bone of the maxilla. Data were obtained on 80 μm slides of the alveolar bone adjacent to the apical third of the right incisor. It was quantified: Calcein Fluorochromes, Active Mineralization Surface, Mineral apposition rate (MAR), Bone volume formed, Mineralized surface, and Bone formation rate. The * indicates significant statistical difference (p<0.05)

The highest bone volume formed occurred in Wistar Losartan with 65650572 μm^3^, showing statistical difference compared with all other groups (p<0.05; Figure 4A).

#### Mineralized surface

Wistar Losartan showed the highest percentage, with 15.34% of mineralized surface, showing statistical difference compared with the other groups (p<0.05, Figure 4B)

#### Active surface of mineralization

Wistar Losartan obtained the highest fluorochrome alizarina biomarking values, showing statistical difference with the Wistar (118235 versus 33189 μm^2^, p=0.011), SHR (118235 versus 37351 μm^2^, p=0.014), and no difference with the SHR group losartan (118235 versus 71638 μm^2^; p=0.1642) (Figure 4C).

#### Bone formation rate

The Wistar Losartan also revealed the highest bone formation rate with 53.9 μm^3^/ μm^2^/day, showing statistical difference compared with both hypertensive animal groups (p<0.05; Figure 4D).

#### Mineral apposition rate

SHR losartan showed higher daily mineral apposition rate compared with Wistar (16.01 versus 10.58 μm, p=0.0141) and SHR (16.01 versus 10.91 μm, p=0.014) and no difference with Wistar Losartan (16.01 versus 13.7 μm, p=0.361) (Figure 4E).

#### Calcein

The highest Calcein fluorochrome biomarker values were in Wistar, with statistical difference when compared with SHR (108578 versus 33052 μm^2^; p=0.0002) and SHR Losartan (108578 versus 50799 μm^2^; p<0.0001) (Figure 4F).

### Histological analysis

**Figure 5:**
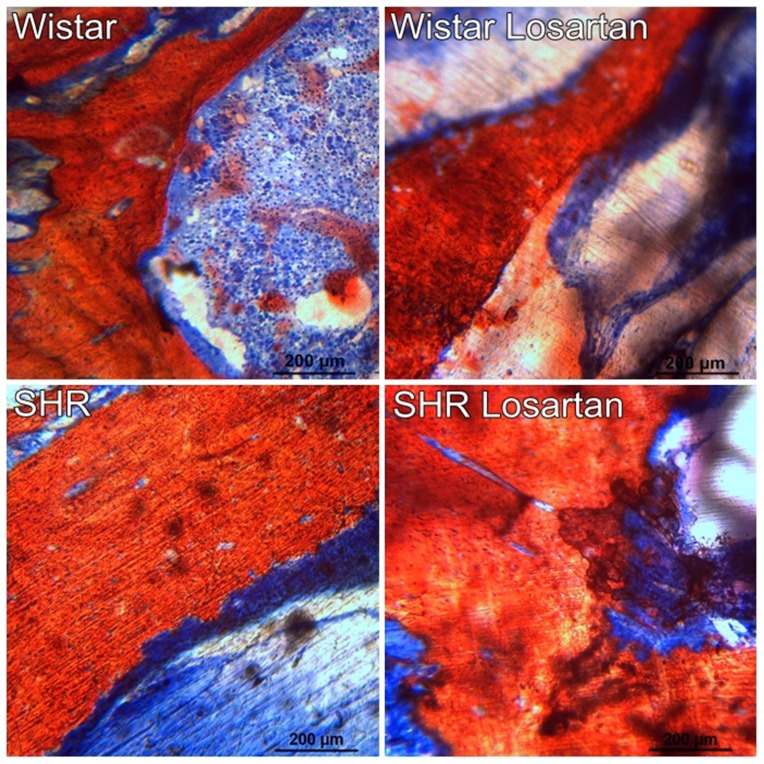
Histology of the maxilla. Histological images of the alveolar bone dynamics in the maxilla for Wistar, Wistar Losartan, SHR, and SHR Losartan. Presence of considerable bone was noted in the medullary compartment. All groups showed similar bone dynamics. Undecalcified thin-ground sections were prepared along the sagittal maxilla axis and Stevenel's blue and red alizarin stained

Figure 5 provides an overview of the bone dynamics in the maxilla. Considerable bone was observed on medullary bone. No obvious differences were visible when comparing all groups. At 10x magnification, medullar bone had undergone bone remodeling. Erythrocytes indicate the presence of the blood vessels. No inflammations signs were observed. Again, these histological findings applied to all groups.

## Discussion

In addition to cardiovascular diseases,^3^ hypertension is associated with changes in bone metabolism.^29^ The renin-angiotensin system modulates inflammation in many conditions. Angiotensin II induces the activation of the extracellular-signal-regulated kinase pathway, having an upregulated RANKL expression, possibly enhancing bone resorption.^22^ This condition is able to decrease alveolar bone density^7^,^30^ and accelerate osteoporosis.^9^ Contrarily, antihypertensive drugs improve bone fracture healing^31^,^32^ and consolidation of bone grafts.^10^ Renin-angiotensin system interferes at vascular and cellular level^33^ in bone metabolism^23^, thus the question arises whether alveolar bone mineralization could be impaired by hypertension. Furthermore, if losartan could improve mineralization by angiotensin II blocks, with consequent increase in calcium supply^34^ and osteoclastogenesis inhibition.^19^ Confirming this hypothesis, this study showed a positive effect of losartan on normotensive animals's alveolar bone dynamics, but not in hypertensive model. Results showed a higher calcium marking in normotensive animals treated with losartan compared with that of other groups. In support of this result, laser confocal microscopy parameters showed high values of bone volume formed, mineralized surface, active surface of mineralization, and bone formation rate in normotensive animals treated with losartan. Results also showed lower mineralized surface in hypertensive animals.

Previous studies corroborate the favorable losartan effect in normotensive animals' alveolar bone dynamics.^10^,^19^,^22^,^23^ One example is the improvement in bone graft consolidation in wistar rats treated with losartan.^10^ Other preclinical studies have shown decreased osteoclastogenesis by losartan, reducing the orthodontic movement^19^ and periodontal bone loss.^22^,^23^ Interestingly, in situations not related to hypertension, such as osteoporosis, this drug could increase bone mineral density in ovarectomized rats' femurs.^21^ Also, other previous findings reveal an angiotensin II increase in hypertension, and its catabolic action on bone metabolism.^8^,^12^ Diversely, estrogen decreased angiotensin II levels.^33^ Thus, osteoprotective effect of losartan was confirmed in fractures of osteoporotic animals, in which losartan increased microcirculation and consolidation of bone fractures.^21^ Additional study reported the ability of losartan to enhance endothelial cell proliferation^34^ and to correct hypertensive patients' arterial structure.^35^ Thus, angiotensin II blocking through losartan has implications beyond simply controlling blood pressure. Regarding vascular activity, its increase in microcirculation and angiogenesis can accentuate cellular activity and subsequent mineral apposition, as in normotensive animals. However, the same effect was not observed in hypertensive animals, which can be explained by a possible exacerbated osteoclastogenic activity in this model as demonstrated in previous studies.^5^,^6^

Regarding the clinical relevance of this study, we highlight the anabolic effect of losartan on bone metabolism in the homeostasis physiological condition. Our findings corroborate a greater mineral deposition in normotensive animals treated with losartan. Although bone dynamics involves a complex mechanism,^26^ we observed two crucial effects of losartan. First, its vasodilatory action possibly increased the plasma calcium supply, and, consequently, bone mineral deposition. Second, its performance on RANKL/OPG system can increase osteoblastic activity and reduce osteoclastogenesis, as validated in previous studies.^6^,^15^,^18^,^22^ For the clinical relevance, studies on the use of losartan to improve osseointegration in normotensive individuals or low bone density condition might be suggested. Also, regarding Implantology, the action of losartan on bone reconstruction with biomaterials can be analyzed to evaluate the occurrence of these benefits. In addition, in Periodontology, it must be investigated whether losartan can prevent or diminish periodontal bone loss. Corroborating our considerations, recent clinical studies showed antihypertensives as responsible for the increased success rate of osseointegrated dental implants.^36^,^37^ Another clinical study shows the reduction in fracture risk in hemodialysis patients treated with antihypertensive drugs.^31^ Additionally, a new study revealed the positive effect of losartan in impaired osseointegration of SHRs.^38^ Considering these results, the need for further studies of the clinical application of losartan is clear. No surgical procedure was performed in this experiment, thus bone dynamics under homeostasis conditions was evaluated, without the influence of a healing stimulus.

Regarding the limitations of this study, determining whether the action of losartan is effective for the improvement in bone dynamics at vascular or cellular level was not possible. Despite the several parameters analyzed, the interpretation of the results was performed considering two specific cellular analysis; extrapolating the results to bone mechanical behavior was not possible. The animal model used only partially reflects the clinical reality and the human bone metabolism. Also, this study did not consider nutritional factors, such as vitamin D and calcium intake levels, which may interfere in bone mineralization. Therefore, future studies should focus on determining how losartan can act on bone metabolism. Efforts should go beyond preclinical studies to check whether alveolar bone density is greater in subjects treated with losartan or other antihypertensives. Finally, future studies should distinguish in which physiological conditions losartan can reverse the inadequate bone metabolism.

In conclusion, losartan can improve bone mineralization in normal physiological conditions, but the same anabolic effect does not occur in hypertension.
